# Combinations of herbs and probiotics as an alternative growth promoter: An *in vitro* study

**DOI:** 10.14202/vetworld.2019.614-620

**Published:** 2019-04-30

**Authors:** Vinsa Cantya Prakasita, Widya Asmara, Sitarina Widyarini, Agnesia Endang Tri Hastuti Wahyuni

**Affiliations:** 1 Study Program of Sains Veteriner, Faculty of Veterinary Medicine, Universitas Gadjah Mada, Yogyakarta 55281, Indonesia; 2 Department of Microbiology, Faculty of Veterinary Medicine, Universitas Gadjah Mada, Yogyakarta 55281, Indonesia; 3 Department of Pathology, Faculty of Veterinary Medicine, Universitas Gadjah Mada, Yogyakarta 55281, Indonesia

**Keywords:** antibiotic growth promoter, feed additive, herbs, probiotic

## Abstract

**Background and Aim:**

Antibiotic growth promoters (AGPs) are added to animal feed to stimulate growth and increase livestock productivity. However, the regular use of antibiotics in animal diets has a considerable contribution to the occurrence of antibiotic resistance in livestock and humans. This study aimed to investigate the feasibility of red ginger (*Zingiber officinale* var. Rubrum), turmeric (*Curcuma domestica*), and wild ginger (*Curcuma xanthorrhiza*), *Lactobacillus acidophilus*, and *Lactobacillus brevis* as an alternative to AGPs.

**Materials and Methods:**

The antibacterial activities and probiotic stimulatory effects of herbs were screened through the disk diffusion method and optical densitometry. The inhibitory ability of probiotics against pathogens was also tested through the disk diffusion method. The adhesion ability of probiotics was tested by mixing the optimal herbal combinations with broiler intestinal epithelial cells (10^5^ cells/ml). The cells were then subjected to Gram staining, and the number of adherent bacteria was calculated.

**Results:**

The test results showed that 3.13% ethanolic wild ginger extract had the highest inhibitory activity against *Salmonella* Enteritidis, followed by ethanolic red ginger extract and aqueous wild ginger extract at the same concentration. The three extracts also supported the growth of *L. acidophilus* and *L. brevis*. Further tests showed that the combination of 3.13% ethanolic red ginger extract had the highest inhibitory activity against *S*. Enteritidis, followed by ethanolic and aqueous wild ginger extract at the same concentration. The three extracts also supported the growth of *L. acidophilus* and *L. brevis*. Further tests showed that the combination of 3.13% ethanolic red ginger extract and 3.13% aqueous wild ginger extract had the best inhibitory effect on the growth of *S*. Enteritidis. The stimulatory effect of the combinations of herbal extract on the growth of *L. acidophilus* (0.18±0.00) and *L. brevis* (0.21±0.01) was better than those of individual extract, positive controls, and the glucose control. *L. acidophilus* and *L. brevis* had a weak inhibitory effect on the growth of *S*. Enteritidis (<6 mm). The adhesion ability of *L. acidophilus* (420.00±28.21) and *L. brevis* (259.33±24.03) was stronger than that of *S*. Enteritidis (202.00±14.00) under treatment with combined extracts.

**Conclusion:**

The tested combinations of herbs and probiotics can adhere to the intestinal tract. Given this characteristic, herb and probiotic combinations may be developed as an alternative to conventional AGPs.

## Introduction

Antibiotic growth promoters (AGPs) are widely added to animal feed to stimulate growth, rapidly increase productivity, and minimize mortality by preventing infections [1]. The use of AGP has a drastic effect on the development and occurrence of antibiotic resistance in animals and humans [2-4]. The World Health Organization and the World Organization for Animal Health have encouraged the health, agriculture, and veterinary sectors to reduce the injudicious use of AGPs [5].

One of the solutions for reducing the use of AGPs is to explore and develop the biodiversity of natural resources of growth promoters. Natural rhizomes such as ginger, turmeric, and wild ginger have numerous beneficial pharmacological effects, such as antimicrobial, anti-inflammatory, antioxidant, anticancer, immunostimulatory, and immunomodulatory effects [6]. The members of Lactobacillus genus are the dominant normal microflora in the poultry digestive tract. *Lactobacillus acidophilus* and *Lactobacillus brevis* are the major species of lactic acid bacteria that provide numerous benefits to poultry health [7].

The utilization of a combination of herbs and probiotics as functional feeds has not been widely studied. Although individual herbs and probiotics are highly valuable, their combinations may enhance their effectiveness and usefulness through synergism. This study aimed to investigate the feasibility of red ginger (*Zingiber officinale* var. Rubrum), turmeric (*Curcuma domestica*), and wild ginger (*Curcuma xanthorrhiza*), *L. acidophilus*, and *L. brevis* as an alternative to AGPs.

## Materials and Methods

### Ethical approval

The Ethics Committee of Universitas Gadjah Mada (UGM), Yogyakarta, Indonesia, has declared that this work satisfies ethical requirements (number: 23.04/III/UN1/LPPT/2018).

### Pathogen and probiotics

The pathogen, *Salmonella* Enteritidis B2664, was obtained from the collection of the Center for Veterinary Research (Bbalitvet, Bogor, Indonesia) and used to evaluate the inhibitory effect of different herbs. *S*. Enteritidis B2664 was isolated from the chicken intestines collected from Bogor Tengah district, Bogor Regency, West Java, Indonesia. The probiotics, *L. acidophilus* FNCC 0051 and *L. brevis* FNCC 0021, were obtained from the collection of UGM Inter-University Center (PAU, Yogyakarta, Indonesia). The pathogens were cultured in Broth Heart Infusion Media (BHI; Merck™), incubated at 37°C for 24 h [8,9]. *L. acidophilus* and *L. brevis* were cultured on de Man-Rogosa Sharpe broth media (MRS; Merck™), incubated at 37°C for 24 h under CO_2_-enriched condition [7,10].

### Herb extract

Rhizomes of red ginger (*Z. officinale* var. Rubrum), turmeric (*C. domestica*), and wild ginger (*C. xanthorrhiza*) were used in this study. The rhizomes were extracted using 96% ethanol and water.

### 96% Ethanolic extracts

Ethanolic extract of red ginger, turmeric, and wild ginger was obtained from the Department of Pharmaceutical Biology, Faculty of Pharmacy, UGM. Extraction was performed through the maceration extraction method. Extracts were prepared at seven different concentrations (100%, 50%, 25%, 12.5%, 6.25%, 3.125%, and 1.67%).

### Aqueous extracts

Dry form of red ginger, turmeric, and ginger were obtained from the Department of Pharmaceutical Biology, Faculty of Pharmacy, UGM. Extraction was prepared by adding sterile distilled water (25% w/v) to dry of rhizomes. The extracts were then stored for 24 h with occasional stirring. The suspensions were centrifuged for 5 min at the speed of 10,000 rpm [11]. Extracts were prepared at five different concentrations (25%, 12.5%, 6.25%, 3.125%, and 1.67%).

### Determination of antibacterial activities and stimulatory effects

#### Diffusion disk test

A modified Kirby-Bauer diffusion disk method was performed to assay the antibacterial activities against pathogens and the stimulatory effects on probiotic growth exerted by the herbs [12]. Suspensions of *S*. Enteritidis and Lactobacillus were spread on Mueller Hinton Agar (MHA; Merck™) and MRS (Merck™), respectively, at a density of 1.5×10^8^ colony-forming unit per milliliter (CFU/mL) [13]. 20 µL of herbal extract was individually added to blank disks (Oxoid™) which were then placed on the surfaces of media. Enrofloxacin disk (ENR 5μg, Oxoid™) was used as positive controls. A disk treated with sterile distilled water was used as negative control. Culture media were incubated at 37°C for 24 h (under CO_2_-enriched conditions for Lactobacillus growth). The diameter of each zone after incubation was measured in millimeters. The clear zone surrounding the disk was used as an index of herbal antibacterial activity against pathogens, whereas the growth zone around the disk represented the ability of herbal extracts to support bacterial growth. All tests were conducted in triplicate.

#### Optical density (OD) test

The minimum inhibitory concentration against *S*. Enteritidis and the maximum growth-promoting concentration for *L. acidophilus* and *L. brevis* of the optimal extracts were determined through the broth microdilution technique in accordance with the Clinical and Laboratory Standards Institute M07-A9 [14] and Clinical Institute Standard Institute M11-A8 [15] with minor modifications. The best herbal extracts were prepared in the first well. Then, two-fold dilutions of samples were prepared with the concentration range 1.56-25% in 96-well microplates. 10 µL of bacterial suspensions with a concentration of 1.5×10^8^ CFU/mL was added into each well. Broth media without extracts were used as the negative controls. Cultured broth media were used as positive controls. The growth of probiotics in different media was compared with that in 1% glucose medium [16]. Microplate was incubated at 37°C for 24 h (under CO_2_-enriched conditions for Lactobacillus growth). The OD of culture media was determined with a microplate reader (655 nm). The test was performed in triplicate.

### Test of the inhibitory effect of L. acidophilus and L. brevis against S. Enteritidis

The well diffusion method performed by Son *et al*. [17] and Chakraborty and Bhowal [18] was used in this test with modifications. *S*. Enteritidis (1.5×10^8^ CFU/ml) was cultured on MHA media through the pour method. Each well was filled with 20 µL aliquots of *L. acidophilus* (10^6^ CFU/ml), *L. brevis* (10^6^ CFU/ml), or 1:1 mixture of *L. acidophilus* (10^6^ CFU/ml) and *L. brevis* (10^6^ CFU/ml). Enrofloxacin disks (ENR 5μg, Oxoid™) were used as positive controls. Media were incubated at 37°C for 24 h. The diameter of the inhibition zone (DIZ, mm) was measured after incubation.

### Adhesion test

The adhesion ability of *L. acidophilus*, *L. brevis*, and *S*. Enteritidis was tested through a method presented by Abdulla *et al*. [19] with modifications. The intestinal epithelial cells of 14-21-week-old broilers (*Gallus gallus domesticus*) were used in this test. Epithelial cells were scraped from the ileum and cecum using a spatula and suspended in 5 ml of phosphate-buffered saline (pH 7.4, Sigma-Aldrich™). The suspension was centrifuged at 1000 rpm for 10 min and washed twice using the same method. Epithelial cells were counted with a hemocytometer to ensure that solutions had cell concentrations of 10^5^ cells/ml.

The adhesion test was performed as follows: First, epithelial cells and bacterial suspensions (1.5×10^8^ CFU/ml) were mixed at the same volume in 1% herbal combination solution and then incubated at room temperature for 60 min. Bacteria that did not adhere to epithelial cells were separated from the suspensions through centrifugation at 2000 rpm for 10 min. The suspension was washed twice at the same speed. Pellets were fixed on glass slides with methanol and stained using Gram stain. The adhesion index was determined by calculating the number of probiotic bacteria that had attached to 50 epithelial cells.

### Statistical analysis

The data from the disk diffusion test were analyzed through one-way analysis of variance and *post* *hoc* Tukey test. The results of OD determination were analyzed through the Kruskal–Wallis and Mann–Whitney tests, whereas adhesion test data were descriptively analyzed. Differences between means were considered statistically significant when p<0.05.

## Results

### Antibacterial activities of herbal extracts

The DIZs surrounding colonies of *S*. Enteritidis under treatment with the ethanolic and aqueous extracts of red ginger extract, turmeric, wild ginger, and their combination were measured in millimeters. The results are shown in Table-1. The highest inhibitory activity against *S*. Enteritidis (21.33±0.58 mm) was exerted by the ethanolic wild ginger extract, followed by ethanolic red ginger extract (14.33±0.58 mm), ethanolic turmeric extract (8.33±0.58 mm), and aqueous wild ginger extract (8.00±0.58 mm). Aqueous red ginger extract and aqueous turmeric extract demonstrated weak inhibitory activities (<6 mm). The positive control, enrofloxacin, presented antibacterial activity, as indicated by the presence of an inhibition zone (12±0.00 mm). By contrast, distilled water, which was used as the negative control, did not show antibacterial activity.

**Table-1:** Antibacterial activities exerted by ethanolic and aqueous herbal extracts against *Salmonella* Enteritidis.

Extract	Diameter inhibition zona^1^ (DIZ, mm)															

	1.56%		3.13%		6.25%		12.50%		25%		50%		75%		100%	
							
	A	E	A	E	A	E	A	E	A	E	A	E	A	E	A	E
Red ginger	ND	7.00±0.00	ND	8.00±0.00	ND	9.00±0.00	ND	12.1±0.29	ND	12.3±0.58	ND	14.3±0.58	ND	13.5±0.50	ND	11.3±0.29
Turmeric	ND	ND	ND	ND	ND	ND	ND	7.17±0.29	ND	8.33±0.58	ND	8.33±0.58	ND	8.33±0.58	ND	7.50±0.50
Wild ginger	8.00±0.29	ND	7.00±0.00	ND	6.50±0.00	8.00±0.00	7.33±0.29	10.67±0.58	7.50±0.50	9.67±0.58	ND	17.00±1.0	ND	21.33±0.58	ND	18.33±0.58
Combination	ND	ND	ND	ND	ND	ND	7.17±0.29	9.50±0.50	7.67±0.58	11.0±0.00	ND	10.33±0.58	ND	10.33±58	ND	10.33±0.58

1 diameter inhibition zone (DIZ, mm) included diameter of disk (6 mm); values are expressed as the means±SD (n=3). A, water extract; E, ethanol extract; ND, not detected: If the DIZ value is <6 mm.

*Significant difference (p<0.05) between the herbal extract and negative control.

The extracts with moderate (6-11 mm) and moderate-to-high antibacterial activity (>11 mm) were further tested through the determination of the OD value of bacterial growth in liquid media enriched with 1% extract (Table-2). The turbidity of the liquid culture of *S*. Enteritidis enriched with 1.56-25% ethanolic wild ginger extract or 3.13-25% aqueous wild ginger extract did not increase. The OD of the liquid culture of *S*. Enteritidis enriched with 1.56% aqueous wild ginger extract increased to 0.14±0.01, which is the same that of the positive control. The culture media enriched with 3.13-25% ethanolic red ginger extract exhibited antibacterial activity against *S*. Enteritidis as evidenced by the lack of an increase in OD value. The OD of the cultured media enriched with 1.56% ethanolic red ginger extract, however, increased (0.07±0.02). This increase was not significant because the value was significantly different from that of the positive control (0.14±0.01).

**Table-2:** Growth of *Salmonella* Enteritidis in media enriched with herbal extracts.

Extract	Optical density (655 nm)					Positive control

	1.56%	3.13%	6.25%	12.5%	25%	
Red ginger ethanol extract	0.07±0.02^a^	0.00±0.00^a^	0.00±0.00^a^	0.00±0.00^a^	0.00±0.00^a^	0.14±0.01
Wild ginger ethanol extract	0.00±0.00^a^	0.00±0.00^a^	0.00±0.00^a^	0.00±0.00^a^	0.00±0.00^a^	0.14±0.01
Wild ginger water extract	0.14±0.01	0.00±0.00^a^	0.00±0.00^a^	0.00±0.00^a^	0.00±0.00^a^	0.14±0.01

a Significant difference (p<0.05) between the herbal extract and positive control.

### Growth stimulatory activities of herbal extracts toward probiotic bacteria

Treatment with ethanolic and aqueous herbal extract did not result in the formation of an inhibition zone around *L. acidophilus* and *L. brevis* colonies. The diameters of the growth zones of each probiotic species around the ethanolic and aqueous extract disks could not be measured because probiotics did not grow with rounded forms. The OD value of *L. acidophilus* culture in MRS broth media enriched with all herbal extracts was higher than those of the positive control (Table-3). The OD value of the growth media enriched with 1.56-6.25% ethanolic red ginger extract, 1.56% ethanolic wild ginger extract, and 1.56% and 6.25% aqueous wild ginger extract was significantly different (p<0.05) from that of the positive and glucose controls. The OD value of the growth media enriched with 12.5% ethanolic red ginger extract, 3.13-12.5% ethanolic wild ginger extract, and 3.13% and 12.5% aqueous wild ginger extract was significantly different from that of the positive control (p<0.05) but was lower than those of the glucose control. On average, the growth of probiotic bacteria in media enriched with 25% herbal extracts was lower than that of probiotic bacteria grown in media enriched with other concentrations of herbal extracts and glucose control.

**Table-3:** Growth of *Lactobacillus acidophilus* in media enriched with herbal extracts.

Extract	Optical density (655 nm)					Positive control^a^	Glucose control^b^

	1.56%	3.13%	6.25%	12.5%	25%		
Red ginger ethanol extract	0.168±0.002^ab^	0.198±0.012^ab^	0.237±0.005^ab^	0.185±0.017^a^	0.135±0.011	0.121±0.006^b^	0.163±0.005^a^
Wild ginger ethanol extract	0.173±0.003^ab^	0.180±0.021^a^	0.168±0.008^a^	0.162±0.004^a^	0.114±0.009^b^	0.121±0.006^b^	0.163±0.005^a^
Wild ginger water extract	0.191±0.006^ab^	0.177±0.010^a^	0.185±0.005^ab^	0.148±0.018^a^	0.124±0.012^b^	0.121±0.006^b^	0.163±0.005^a^

a Significant difference (p<0.05) between the herbal extract and positive control,

b Significant difference (p<0.05) between the herbal extract and glucose control

The OD value of *L. brevis* grown in media enriched with 1% herbal extract is presented in Table-4. The OD value of media enriched with 1.56-12.5% ethanolic red ginger extract, ethanolic wild ginger extract, and aqueous wild ginger extract was greater than those of the positive and glucose controls. Growth media enriched with 6.25% ethanolic red ginger extract showed the highest OD value and significantly superior stimulatory effects on the growth of *L. brevis* (p<0.05) than the positive and glucose controls. However, the OD values of media enriched with 25% herbal extracts were lower than those of the control.

**Table-4:** Growth of *Lactobacillus brevis* in media enriched with herbal extracts.

Extract	Optical density (655 nm)					Positive control^a^	Glucose control^b^

	1.56%	3.13%	6.25%	12.5%	25%		
Red ginger ethanol extract	0.171±0.011^ab^	0.183±0.005^ab^	0.197±0.008^ab^	0.142±0.011	0.106±0.014^b^	0.123±0.022^b^	0.152±0.004^a^
Wild ginger ethanol extract	0.151±0.010^ab^	0.177±0.003^ab^	0.171±0.002^ab^	0.157±0.008^a^	0.113±0.015^b^	0.123±0.022^b^	0.152±0.004^a^
Wild ginger water extract	0.185±0.009^ab^	0.176±0.001^ab^	0.163±0.011^a^	0.143±0.015	0.075±0.011^ab^	0.123±0.022^b^	0.152±0.004^a^

a Significant difference (p<0.05) between the herbal extract and positive control,

b Significant difference (p<0.05) between the herbal extract and glucose control

### Growth stimulatory activities of the combination of herbal extracts toward pathogens and probiotics

Previous test results showed that 3.13% ethanolic red ginger extract, 3.13% ethanolic wild ginger extract, and 3.13% aqueous wild ginger extract are the optimal extracts for inhibiting the growth of *S*. Enteritidis and for stimulating the growth of *L. acidophilus* and *L. brevis*. The extracts were divided into two extract combinations: Extract combination 1, which comprised ethanolic red ginger and wild ginger extracts, and extract combination 2, which comprised ethanolic red ginger and aqueous wild ginger extracts.

The cultures of *L. acidophilus* and *L. brevis* in media enriched with extract combination 1 and 2 showed the highest OD values (Table-5). Extract combination 2 was able to support the growth of *L. acidophilus* and *L. brevis* and also had better antibacterial activity against *S*. Enteritidis than extract combination 1 (p<0.05). These results demonstrate that herbal extracts can stimulate the growth of probiotics and have antibacterial activity against pathogens as mentioned by Zhou *et al*. [20] and Zadeh and Kor [21].

**Table-5:** Growth of *L. acidophilus*, *L. brevis*, and *S*. Enteritidis in media enriched with herbal extracts.

Herbal combination	Optical density (655 nm)		

	*L. acidophilus*	*L. brevis*	*S. Enteritidis*
Extract combination 1	0.23±0.01^ab^	0.20±0.00^b^	0.11±0.04^ab^
Extract combination 2	0.18±0.00^b^	0.21±0.01^ab^	0.00±0.00^a^
Positive control^a^	0.17±0.00	0.18±0.02	0.27±0.01
Glucose control^b^	0.16±0.04	0.15±0.04	

a Significant difference (p<0.05) between the herbal extract and positive control,

b Significant difference (p<0.05) between the herbal extract and glucose control. *L. acidophilus*=*Lactobacillus acidophilus*, *L. Brevis*=*Lactobacillus brevis*, *S*. Enteritidis=*Salmonella* Enteritidis

### Inhibitory activities of L. acidophilus and L. brevis against S. Enteritidis

Probiotics and their combinations exerted a weak inhibitory effect on *S*. Enteritidis, as indicated by the presence of a DIZ of <6 mm.

### Adhesion ability of L. acidophilus, L. brevis, and S. Enteritidis to intestinal epithelial cells

**Figure-1:**
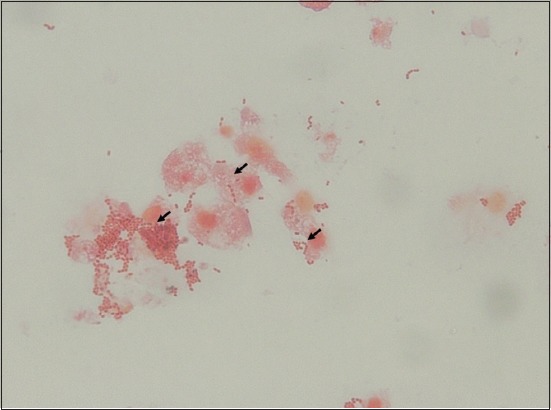
Ability of *L. acidophilus* (black row) to adhere to intestinal epithelial cells as observed through Gram staining (1.000×).

An adhesion test was conducted to determine the adhesion ability of *L. acidophilus, L. brevis*, and *S*. Enteritidis to the intestinal epithelial cells of broilers. The adhesion ability of each bacterial isolate to epithelial cells is presented in Table-6. The adhesion ability of each bacterial species differed. The highest adhesion ability was exhibited by *L. acidophilus* (Figure-1), followed by *L. brevis* and *S*. Enteritidis.

**Table-6:** Ability of *L. acidophilus*, *L. brevis*, and *S.* Enteritidis to adhere to 50 cells.

Isolate	Number of bacteria attached to 50 cells
*L. acidophilus*	420.00±28.21
*L. brevis*	259.33±24.03
*S.* Enteritidis	202.00±14.00

*L. acidophilus*=*Lactobacillus acidophilus*, *L. Brevis*=*Lactobacillus brevis*, *S.* Enteritidis=*Salmonella* Enteritidis

## Discussion

Ethanolic wild ginger extract (1.56-25%) exhibited the highest inhibitory activity against *S*. Enteritidis, followed by ethanolic red ginger (3.13-25%) and aqueous wild ginger (3.13-25%) extracts. Wild ginger extract contains xanthorrhizol (XNT), an antimicrobial terpenoid compound that is absent from other Curcuma rhizomes [22]. Although the exact mechanism underlying the antimicrobial activity of XNT remains unclear, the suppression of nuclear factor kappa-B (NF-kB) and mitogen-activated protein kinase (MAPK) by XNT will stimulate immune responses [23]. XNT may account for higher inhibitory power of wild ginger than that of turmeric and red ginger. The essential oils, gingerol and zingiberene, which are phenol derivatives, are the main antimicrobial compounds of red ginger [24,25]. Test results showed that the antibacterial activity of extracts with an ethanolic base is better than that of extracts with an aqueous base because the antibacterial compounds present in ethanolic extracts are more exposed than those in aqueous extracts [26].

The extracts were able to support the growth of *L. acidophilus* and *L. brevis*, and all three extracts inhibited *S*. Enteritidis growth. Zhou *et al*. [20] and Molan *et al*. [27] reported that polyphenols in herbal extracts exert their high antioxidant activity against free radicals and oxidative stress generated by metabolic activity by providing a microaerophilic environment for the growth of probiotic bacteria. In addition, the carbohydrate content of herbal extract can be a good substrate for probiotic growth [28]. Further tests showed that the combination of 3.13% ethanolic red ginger extract and 3.13% aqueous wild ginger extract had an inhibitory effect on *S*. Enteritidis (0.00±0.00) and had better growth-stimulating effects on *L. acidophilus* (0.18±0.00) and *L. brevis* (0.21±0.01) than individual extracts and positive and the glucose controls.

*L. acidophilus, L. brevis*, and even the combination of both bacteria had a weak inhibitory effect on *S*. Enteritidis (<6 mm). Son *et al*. [17] and Fuller [29] reported that 10^7^-10^8^ CFU/g Lactobacillus effectively suppressed the growth of pathogenic bacteria by decreasing the acidity associated with lactic acid production. In the present study, the adhesion ability of *L. acidophilus* (420.00±28.21) and *L. brevis* (259.33±24.03) to intestinal epithelial cells was greater than that of the pathogenic bacteria species (202.00±14.00). *L. acidophilus* and *L. brevis* may prevent pathogens from adhering to the intestinal tract by binding to intestinal mucus. This hypothesis is supported by Ma *et al*. [30] who reported that the intensive binding of probiotics to intestinal mucus is indicative of low pathogen adhesion to the intestinal tract.

## Conclusion

The combination of herbal and probiotics can adhere to the intestinal tract and may thus be developed as an alternative to AGP.

## Authors’ Contributions

VCP: Microbial and herbal preparation, doing all tests, and statistical analysis. WA: Microbial (probiotics and pathogen) analysis. SW: Epithelial cell sampling and adhesion test analysis. AETHW: Main ideas, research design, and analysis. All authors read and approved the final manuscript.
